# Supplementary material for: The interactome of intact mitochondria by cross-linking mass spectrometry provides evidence for coexisting respiratory supercomplexes
Source: Mol Cell Proteomics. 2017 Dec 8;17(2):216–32. doi: 10.1074/mcp.RA117.000470 (PMC5795388; doi:10.1074/mcp.RA117.000470)
Supplement: Supplemental Data [file supp_RA117.000470_133922_0_supp_23978_fzffwf.zip › spectra_annotation/mito_spectra_annotation/469-2-11-1-5-1.pdf]

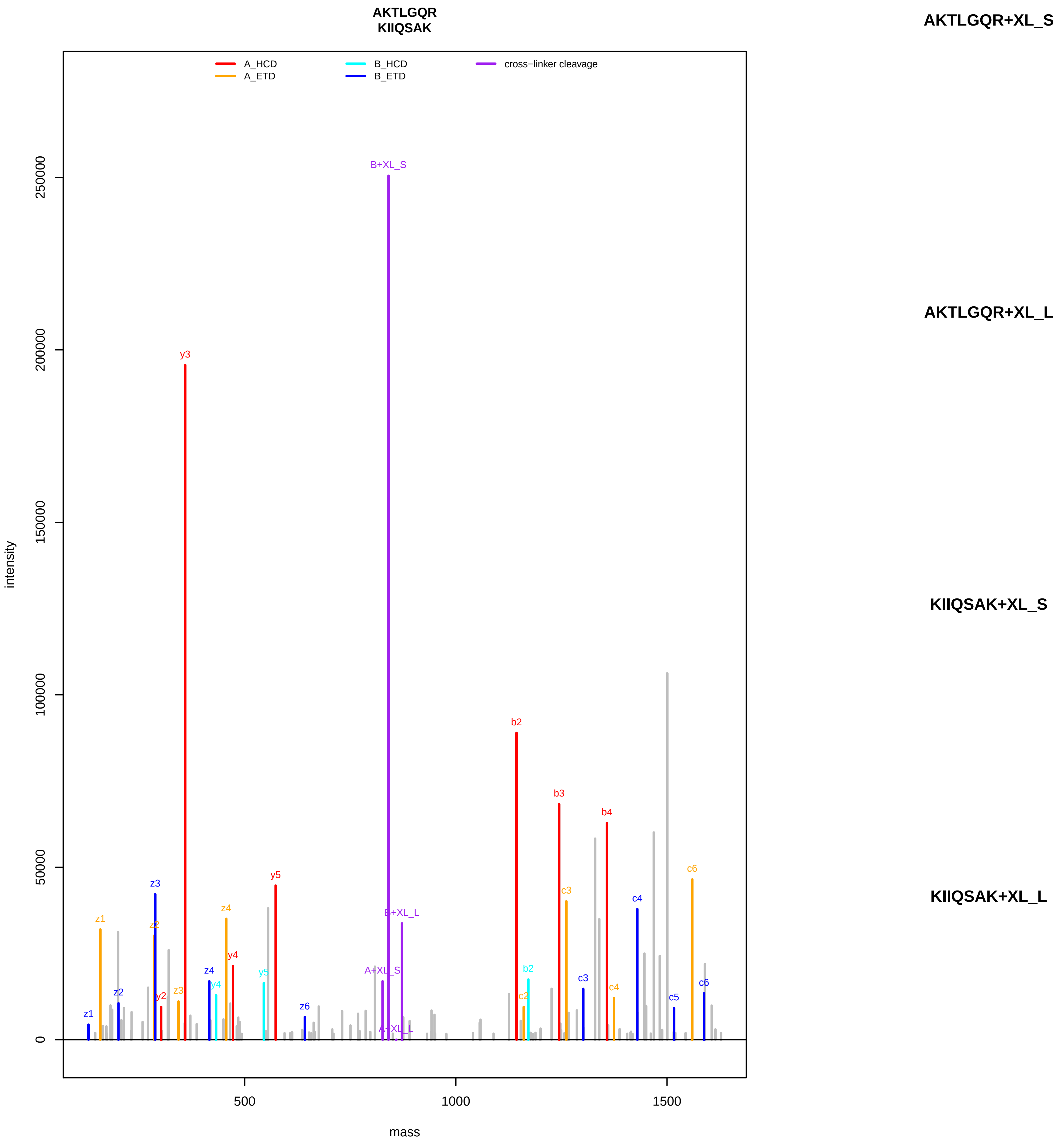
AKTLGQR
KIIQSAK
A_HCD
A_ETD
B_HCD
B_ETD
cross−linker cleavage
intensity
mass
0
50000
100000
150000
200000
250000
500
1000
1500
B+XL_S
y3
b2
b3
b4
c6
c3
c4
c2
b2
y5
z1
z2
z3
z4
y4
y5
z6
c3
c4
c5
c6
y2
z3
y4
z4
B+XL_L
A+XL_S
A+XL_L
AKTLGQR+XL_S
AKTLGQR+XL_L
KIIQSAK+XL_S
KIIQSAK+XL_L
